# Co-designing models for the communication of genomic results for rare diseases: a comparative study in the Czech Republic and the United Kingdom

**DOI:** 10.1007/s12687-022-00589-w

**Published:** 2022-05-06

**Authors:** Alessia Costa, Věra Franková, Glenn Robert, Milan Macek, Christine Patch, Elizabeth Alexander, Anna Arellanesova, Jill Clayton-Smith, Amy Hunter, Markéta Havlovicová, Radka Pourová, Marie Pritchard, Lauren Roberts, Veronika Zoubková, Alison Metcalfe

**Affiliations:** 1 Engagement and Society, Wellcome Connecting Science, Hinxton, CB10 1SA Cambridgeshire UK; 2 Faculty of Nursing, Midwifery and Palliative Care, King’s College London, London, SE1 8WA UK; 3 Department of Paediatrics and Inherited Metabolic Disorders, Charles University, First Faculty of Medicine and General University Hospital, Prague, Czech Republic; 4 Institute for Medical Humanities, Charles University, First Faculty of Medicine, Prague, Czech Republic; 5 Department of Biology and Medical Genetics, Charles University, Second Faculty of Medicine, and University Hospital Motol, Prague, Czech Republic; 6 Genomics England, London, EC1M 6BQ UK; 7 Manchester Centre For Genomic Medicine, University of Manchester, St Mary’s Hospital, Manchester, M13 9WL UK; 8 Česká asociace pro vzácná onemocnění (ČAVO), Rare Diseases Czech Republic, Bělohorská 19, Praha 6, 169 00 Czech Republic; 9 Division of Evolution and Genomic Sciences School of Biological Sciences, University of Manchester, Manchester, M13 9PL UK; 10 Genetic Alliance UK, London, EC2A 4NE UK; 11 Syndromes Without A Name (SWAN UK), London, EC2A 4NE UK; 12 Zinc Ventures, London, UK

**Keywords:** Genomic testing, Genetic services, Rare diseases, Experience-based co-design, Patient involvement

## Abstract

The communication of genomic results to patients and families with rare diseases raise distinctive challenges. However, there is little evidence about optimal methods to communicate results to this group of service users. To address this gap, we worked with rare disease families and health professionals from two genetic/genomic services, one in the United Kingdom and one in the Czech Republic, to co-design that best meet their needs. Using the participatory methodology of Experience-Based Co-Design (EBCD), we conducted observations of clinical appointments (*n*=49) and interviews with family participants (*n*=23) and health professionals (*n*=22) to gather their experience of sharing/receiving results. The findings informed a facilitated co-design process, comprising 3 feedback events at each site and a series of meetings and remote consultations. Participants identified a total of four areas of current service models in need of improvement, and co-designed six prototypes of quality improvement interventions. The main finding was the identification of post-test care as the shared priority for improvement for both health professionals and families at the two sites. Our findings indicate the need to strengthen the link between diagnostics (whether or not a pathogenic variant is found) and post-test care, including psychosocial and community support. This raises implications for the reconfigurations of genomic service models, the redefinition of professional roles and responsibilities and the involvement of rare disease patients and families in health care research.

## Introduction

The communication of genomic results to rare disease patients and their families presents distinctive challenges due to both the mode of testing and the needs of this particular group of service users. As opposed to testing targeting single genes or gene ‘panels’ of limited size, genomic testing involves examination of a much larger amount of an individual’s DNA, meaning there is wider room for uncertainty about possible findings and their implications for the individual family. This means that established challenges related to the disclosure of genetic information not only remain relevant in the context of genomics, but are likely to be exacerbated (Eisler et al. 2016; Dheensa et al. 2017; Metcalfe et al. 2011; Hallowell et al. 2006). Moreover, genomic testing also raises novel and unique issues for genetic counselling. The communication of inconclusive and uncertain results has been identified as an area of concern (Clift et al. 2020; Mighton et al. 2021; Skinner et al. 2018; Fenton et al. 2020; Bartley et al. 2020, 2021; Han et al. 2017). The limitations for informed consent and pre-test counselling have also been highlighted, as the uncertainty surrounding genomic results means it is increasingly difficult to ensure that families can be prepared for the testing possible outcomes (Horton and Lucassen 2019; Samuel et al. 2017; Newson et al. 2016).

Compounding these general issues are the unique challenges associated with rare disease diagnostics. The rarity and heterogeneity of rare diseases, and their chronic and progressive nature can have a significant psychosocial impact on families. Genomic testing is crucial to ensure families can receive a timely and accurate diagnosis, but can only alleviate the burden of living with a rare disease (Rosell et al. 2016). With or without a diagnosis, families often face significant financial, social and emotional challenges, affecting their quality of life and their capacity to access high quality care (Pelentsov et al. 2015; von der Lippe et al. 2017). However, there is scarce evidence on what model of genomic services might best meet patients’ and families’ needs. Understanding the implications of these issues will be crucial to maximise the benefits of genomic sequencing as this rapidly moves from research into routine care (Stark et al. 2019; 100000 Genomes Project Pilot Investigators 2021),

To help collect cross-national responses to these issues, we worked with families of patients with rare diseases and health professionals to co-design communication models based on their experience of sharing/receiving genomic results. The study was conducted as part of the EU Horizon 2020 research project Solve RD (http://solve-rd.eu) and was set in two genetic/genomic services in the United Kingdom (UK) and in the Czech Republic. We used the participatory methodology of Experience-Based Co-Design (EBCD), which draws on design thinking to enable service users to become integral to the process of service design and quality improvement (Bate and Robert 2007; Robert et al. 2021). The approach was initially developed and piloted in a Head & Neck Cancer service in an English acute hospital in 2004–2005, and has subsequently been implemented in over a hundred different services in approximately ten countries, as well as being used to help develop complex interventions (Donetto et al. 2014; Robert et al. 2021). Using EBCD, we worked with families and health professionals at the two participating services to explore their experience, identify areas of current models in need of improvement and co-design quality improvement interventions.

In this article, we present and compare the EBCD process and outcomes at the two sites. We focus in particular on the issue of post-test care, as this was the key priority for improvement that was identified by both health professionals and families at both sites. The findings can be adapted to improve the communication of genomic results in other countries and health care settings.

## Methods

### Setting and participants

The participating services were chosen to include Solve RD partners representative of different European countries. Both are part of major university teaching hospitals and active in the European Reference Network (ERN) on Intellectual disability, TeleHealth, Autism and Congenital Anomalies (ITHACA)^1^. At the Czech site, clinical geneticists were responsible for offering the test to families and communicating the results. Although genetic counselling is not recognised as an independent profession, the service is bound by specific genetic legislature stipulating that pre-/post-test genetic counselling by clinical geneticist must be provided in diagnostic cases where severe and/or actionable results are expected. At the UK site, both clinical geneticists and genetic counsellors were involved in consenting patients and communicating results, but the former were mainly responsible for genomic testing for rare disease diagnostics. At the time of the study, genomic testing was integrated into routine clinical care in both countries. In the Czech Republic, the delivery of genomic testing was led by genetic health professionals, whereas in the UK, non-genetic specialists were also involved in ordering and discussing genomic testing.

The recruitment of health professionals was informed by discussion with senior staff and reflected the different organisational arrangements of the two services. At both sites, we invited clinical geneticists, and clinical scientists, for their role in drafting of laboratory reports and deciding which results are fed back. We also recruited genetic counsellors (at the UK site) and nurses (at the Czech site), for their involvement with families at different moments in the family journey through the services. Participants were free to step in and out of the study at different stages (see Table 1 for participant numbers throughout the process).

**Table 1:** Participants’ details

Study phase	Patient/family			Health professionals		
Interviews	*Family member*	CZ	UK		CZ	UK
	Mother	3	5	Clinical geneticist	7	5
	Father	0	1	Genetic counsellor	N.A.	4
	Joint interview (both parents)	4	2	Scientist	2	2
	Other (grandmother)	2	0	Nurse	1	0
				Non-genetic specialist	0	1
	*Type of results*					
	Diagnosis	5	6			
	VUS	0	1			
	Null	4	1			
Feedback events	*Family event*	12	8	*HP event*	22	12
	Mother	3	4	Clinical geneticist	13	4
	Father	1	2	Genetic counsellor	N.A.	8
	Grandmother	1	N.A.	Scientist	6	0
	Patient	2	1	Nurse	2	0
	FAG member	5	1	Genetic anthropologist	1	N.A.
Joint event	*Family participants*	11	8	*HP participants*	17	9
	Mother	4	3	Clinical geneticist	12	4
	Father	2	2	Genetic counsellor	N.A.	5
	Other (grandmother)	0	0	Scientist	3	0
	Patient	1	1	Nurse	1	0
	FAG member	4	2	Genetic anthropologist	1	N.A.
Co-design meetings	Mother	0	1	Clinical geneticist	5	3
	Father	1	0	Genetic counsellor	N.A.	4
	FAG member	2	4			
	Patient	0	1			

To represent a variety of views, we invited families who had received different types of results, including where a diagnosis was confirmed based on the detected variant(s), where variant(s) of uncertain significance (VUSs) were detected that did not lead to a diagnosis and where the results were null. Diagnoses could include confirmation of clinical diagnoses or newly identified diagnoses, including ‘ultra-rare’ diagnoses that have only recently been reported and described in the clinical literature. Eligibility criteria included (i) families of patients with potential syndromic diagnoses with a genetic aetiology; (ii) who had received results from various genome analyses comprising either exome or genome sequencing; (iii) who received results no longer than three years prior to the study, to ensure the findings were relevant and timely. Families were recruited through the participating genetic services and, at the UK site, through the charity Syndromes Without A Name (SWAN).

### EBCD process

**Fig. 1:**
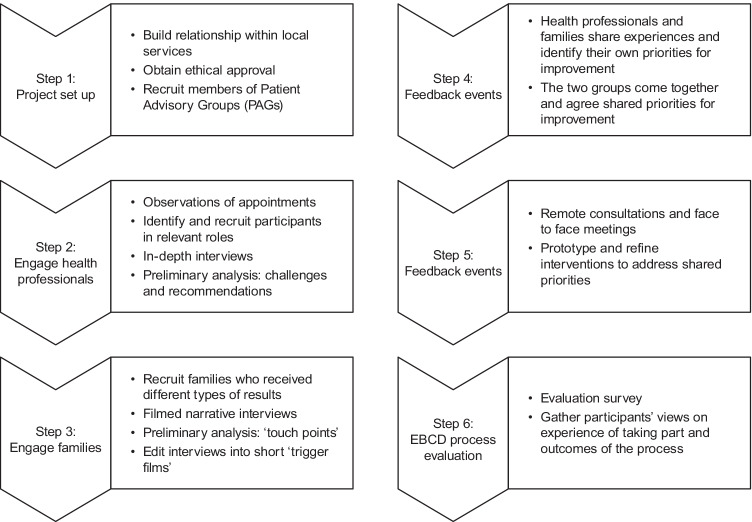
Study diagram

The study took place between April 2018 and January 2020 following the 6-stage EBCD process (see also Figure 1).

#### Setting up the project (April–August 2018)

Ethical approval was obtained from the Ethical Committees of the respective services and the UK Health Research Authority. At both sites, we set up a Family Advisory Group in collaboration with local patients’ organisations (i.e. Rare Diseases Czech Republic and Syndromes Without A Name). The groups comprised rare disease patients and families recruited through the organisations. They were consulted regularly to ensure patients’ and families’ views were reflected in the different phases of the study.

#### Engaging health professionals (September 2018–February 2019)

The researchers (AC, VF) conducted non-participant observations of clinical appointments to document the family journey through the respective services. Written material was displayed at the reception to inform families of the presence of the researcher, and health professionals introduced the study and asked for family consent prior to each consultation. The researchers sat quietly at the back of the room and took note of the information that was shared with families and of the family-clinician interactions. The researchers also conducted semi-structured interviews with health professionals to explore their experience and approaches, as well as their views on what worked well and what could be improved at the services. The interviews were audio-recorded and professionally transcribed and, in the case of the Czech materials, translated into English by a professional translator. Data was analysed according to established principles of thematic analysis (Corbin and Strauss 1990; Glaser et al. 1999) and contextualised with reference to the observational notes. Data from each site was first analysed independently by the researchers, and emerging themes were iteratively compared and discussed with the larger team.

#### Engaging families (February–April 2019)

The researchers conducted narrative interviews with family members using a semi-structured schedule exploring different aspects of their experience, including their expectations about results, how these were communicated to them, what happened afterwards and how the process could be improved. The interviews were filmed; audio files were fully transcribed and translated into English and selected clips were edited into two short films, one per site, summarising key ‘touchpoints’. These represent salient themes, both positive and negative, that had emerged from the interviews. They reflect not only the frequency with which a theme is identified but also, and most importantly, its emotional impact as described by participants (Bate and Robert 2007). The same analytical approach used for health professional interviews was applied to this data in order to identify the touchpoints. Interviewees had the opportunity to review their own clips before the film was shared with other participants and all provided written consent to the public use of their images.

#### Feedback events (April–June 2019)

At each site, we held three events with the following order: (i) health professionals, (ii) families and (iii) the two groups together. The event with health professionals lasted 120 min and was open to all members of staff at the respective service, including those who had not been previously involved in the study. After sharing a summary of the challenges and recommendations for improvement identified through preliminary analysis of the interviews, we invited participants to use these preliminary findings to discuss in small groups what worked well and what could be improved. During the plenary discussion, feedback from each group was clustered together into broad thematic areas using Post-It to provide a structured visual representation of possible areas for improvement. Finally, participants were asked to vote which improvement areas they wanted to prioritise for further discussion with families.

The family feedback event lasted 150 min. Participants watched the edited film and were asked to provide feedback on each touchpoint, as well as to suggest any new or emerging themes. The facilitated discussion focused on identifying priorities for improvement. First, we run a prioritisation exercise using non-cumulative voting, to rank the different touchpoints from the film. Then, we carried out a rapid prototyping exercise (IDEO 2015: 85) to translate each touchpoint into one or more service areas in need of improvement.

At the final event, which lasted 150 min, participants had the opportunity to discuss each other’s views—triggered by the short films on family experience. The outcomes from the previous events were reviewed to identify convergences between health professionals’ and families’ priorities. Participants were then invited to select shared priorities through cumulative voting. Discussion at the events was facilitated by members of the research team with experience of moderating similar events (AA and GR) and documented by the researchers. Researcher notes were included for analysis in this article.

#### Co-design meetings/consultations (June–November 2019)

For each of the final priority identified at the joint events, we distilled core design principles. These are the core elements of the desired solutions which guide the different iterations of the co-design work (IDEO 2015). The core design principles were derived from the interviews and discussions at the events. They were shared with participants following the events, along with practical resources to draw inspiration from for the co-design work (e.g. models of existing interventions). Over a series of face-to-face meetings and email consultations, we invited participants to use the principles to iteratively develop and refine prototypes of interventions to address the priorities selected.

#### Process evaluation (April–June2019)

After each feedback event, we collected participants’ anonymous feedback using evaluation surveys from previous EBCD projects (Locock et al. 2014). The surveys contained Likert-scale questions and open-ended questions to gather participants’ feedback on different aspects of the EBCD process and outcomes, including their overall impressions and feelings about the events, content of edited film and selected priorities, as well as their suggestions for future improvement. Responses were analysed thematically and researchers’ notes from the events were used to complement the analysis.

## Results

Ten qualitative themes and seven ‘touch points’ emerged from the comparative analysis of health professionals’ and families’ interviews (see Tables 2 and 3 for details). These translated into four priorities for improvements, including one (post-test care) that was shared across the two sites and three that were specific to the respective service (see Table 4). A total of six prototypes of quality improvement interventions were co-designed to address these priorities (see Table 5). In the remainder of this article, we focus on post-test care, as the one area for improvement identified by health professionals and families at both sites. We illustrate how post-test care emerged as an area of particular concern for both groups of participants, and describe the key components of the relevant quality improvement interventions. We conclude with a reflective evaluation of the EBCD process at the two sites.

**Table 2:** Themes from interviews with health professionals and discussion at the events

Czech site	UK site
**Post-test care:** need to follow up with families after results have been shared, and recommendations for improvements in this area.	
**Telehealth**: improve accessibility to facilitate communication with families, including via email and/or digital consultation.	
**Multidisciplinary approach**: improve collaboration with non-genetic specialties as well as with allied health care professions (e.g. integrated service models).	
**Education:** about genomics in general and rare disease in particular, particularly among non-genetic specialties and in collaboration with patient organisations.	
**Counselling skills**: psychosocial support on challenging aspects of the family journey (e.g. expectation management, valuing negative results, managing feelings of guilt)	
**Lab reports**: accessibility of language and content of the reports for families and non-genetic professionals.	
**Family-facing educational and information materials** (e.g. improvements to service website)	**Resources** (e.g. workforce shortages, commissioning)
**Service environment** (e.g. wheelchair access, a suitable waiting room, a feeding and changing room for babies and toddlers)	**IT and data sharing** (e.g. patient database)

**Table 3:** Touch points from family interviews

Czech site	UK site
**Personal utility**: benefits families identified, including but not limited to the clinical utility of results (e.g. psychological benefits, benefits to other family members and future patients)	
**Making sense**: the emotional impact of results and information overload at the consultation meant that time was needed to process the implications of the results.	
**Unmet needs**: following the communication of results families often reported having unanswered questions and experiencing challenges in using the new information to improve their care, even when a diagnosis was confirmed.	
**Feelings of guilt and blame**: families’ sense of responsibility about causing the patient’s disability and/or passing on the conditions, which could be induced and/or exacerbated by the results	**Communication at the point of testing**: lack of openness and transparency about the reasons for testing, the different types of possible results and the impact on family’s expectations.
**Service environment**: insufficiently spacious offices for large families, lack of barrier free-access and child-friendly spaces.	**Communication about availability of results**: issues related the communication to inform families that the results are ready, including lack of notice, provision of impartial information and/or long waiting times for appointment, lack of consultation on family preferences.

**Table 4:** Priorities for improvement

Czech site	UK site
Health professional priorities	
Post-test care: follow up with families after results have been shared	Post-test care: facilitate communication after results have been shared (e.g. telehealth)
Family-facing educational and information materials: provide resources and content of the service website	Multidisciplinary collaboration: information that can be used by non-genetic professionals (e.g. at the point of testing)
Lab reports: improve accessibility by and utility for families	Lab reports: clear and standardised reports to improve accessibility by non-genetic professionals
Family priorities	
Post-test care: follow-up consultation	Communication at the point of testing: transparency and expectation management
Psychosocial support: involvement of psychologist and/or social worker at results delivery	Post-test care: support and advice after results are shared
Information provision	Post-test care: named point of contact
Manage feelings of guilt and blame	Communication about results availability
Improvement of service environment	Multidisciplinary care: better coordination between genetic and non-genetic professionals
Shared priorities	
Follow-up consultation	Communication at the point of testing: transparency and expectation management
Managing feelings of guilt and blame	Named point of contact for follow up
Environmental improvements

**Table 5:**
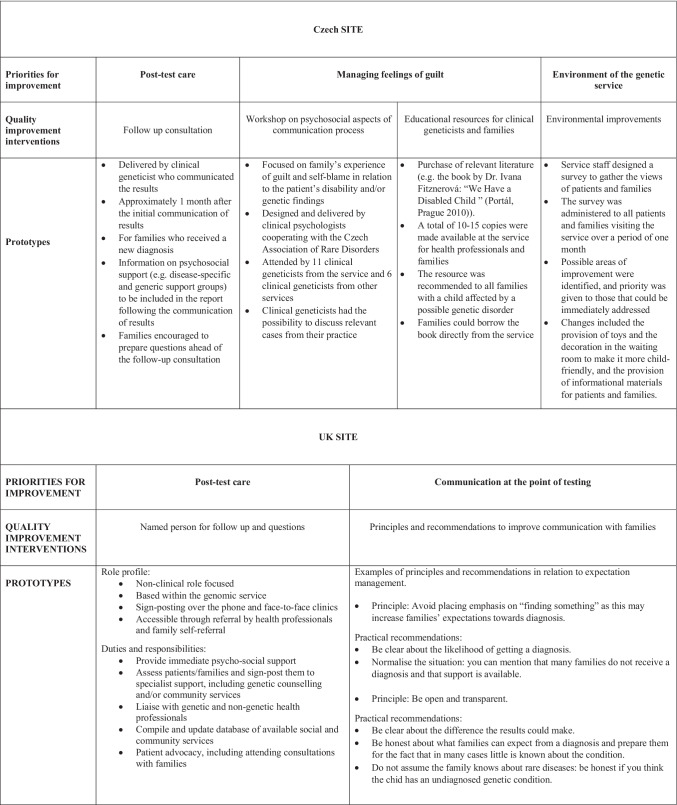
Quality improvement interventions at the two sites

### Observational fieldwork

We conducted non-participant observations of forty-nine appointments (*n*=10CZ; *n*=39UK) with 13 different genetic health professionals (*n*=6CZ; *n*=7UK). Findings from observations were in line with evidence from recent research showing that the communication process tends to be clinician-led (Sanderson et al. 2019) and that there can be a mismatch between families’ priorities and the information shared at the consultation (Watnick et al. 2021).

Conversations about testing and consent took place over the course of first appointments; these lasted up to 45 min at the UK site and up to 75 min at the Czech site. Families were often not clear about the reason why they had been referred to genetics and the implications of testing needed to be explicitly articulated. At both sites, families were routinely informed of the different types of possible results, including the meaning of VUS and negative results. They were asked to sign an informed consent form detailing this information and were provided a copy to take home for their records. We did not observe families receiving other sources of information (e.g. leaflets) or being signposted to other sources of support (e.g. generic patient support groups) at this stage.

Families were regularly informed of the average turnaround time for the results, and at times expressed surprise at the longer waiting time compared to other medical tests. Once the results were available, families were invited via letter or phone call for an appointment at the genetic service. The amount and type of information disclosed at this stage, including whether families, were informed that the appointment was to discuss results and/or if they received any details about the variant(s), was decided on a case-by-case basis and could vary greatly depending on family circumstances and the approach of individual health professionals. However, families were not routinely consulted about their preferences when consenting to the test.

At the Czech site, all types of results were discussed during face-to-face appointments; at the UK site, new diagnoses were communicated in person, negative results were normally shared via letter and decisions about VUS were taken on a case-by-case basis. The length of ‘result appointments’ (up to 30 min at the UK site and up to 50 min at the Czech site) was on average shorter than for the first appointments. Topics normally discussed included: an explanation of the phenotypic features associated with the variant; the mode of rare disease inheritance and any implications for the proband’s and other family members’ reproductive choices; any implications for treatment and new medical referrals; next steps, including future appointments to review the case. The main difference in the style and content of individual consultations concerned the explanation of the molecular diagnosis: a majority of health professionals provided some background information on genes and disease mechanisms as a way to explain the result before discussing the implications for the family; other health professionals did not share this type of information unless asked.

Health professionals at both sites used visual aids when possible, including graphs about the prevalence of phenotypic features, pictures of other patients or images of genes and cells. Disease-specific information materials, however, were scarce, especially in the case of new variants. Where appropriate, images or data from scientific articles were shared at the consultation and a copy was provided for families to take home. The resources, however, were not easily accessible due to the highly technical information and the lack of translation into Czech language.

Overall, the consultation tended to be clinician-led and families remained silent for most of the time. Health professionals explicitly asked families if they had any questions. Families’ questions usually concerned the implications of results, particularly for treatment options, changes in clinical care and access to services and support. In many cases, these questions seemed to reflect families’ pre-existing concerns and long-standing needs rather than elaborating on the information that was shared during the consultation.

### Health professionals’ interviews and feedback events

Twenty-two health professionals took part in the interviews (*n*=10CZ; *n*=12UK) and thirty-four attended the feedback event (*n*=22CZ; *n*=12UK). The improvement areas identified by health professionals were largely similar across the two sites and resulted in a total of six priorities (see Table 2 for a summary of the comparative analysis). At both sites, participants acknowledged the emotional impact of the results, whether or not a diagnosis was confirmed, and the importance to value negative and inconclusive results. In the case where a diagnosis was confirmed, it could be challenging to go through the new information while also making space for families to process the emotional impact of the results:

I take care not to talk too much during the appointment so that they [families] have a chance to ask questions but they are shocked, I believe, and probably need to process it….I feel that the people are usually only sitting there and listening. They have a chance to ask questions but they are shocked, and I get to see the feedback only after some time, i.e. what the impact was and everything else. (Czech clinical geneticist)

Health professionals commented that the dynamic during the consultation was such that families’ attention was directed at following the clinician’s explanation, often with little opportunity to consider personal questions. They suggested that when the results revealed new information about the condition, these should be delivered ‘in two stages’:

Sometimes we’re in the situation where we’re giving a new diagnosis anyway, which is sometimes hard for families. And at a first appointment I find it quite difficult to also then dive in and say, be very gloomy about future prognosis. So what I would normally say is ‘this is what we know at the moment about this condition, you'll have lots of questions, what I suggest is over the next couple of months you think up all those questions and then we’ll bring you back’. So often we give the diagnosis in two stages: one is giving the news and the basic information and the name of the syndrome and things and then I might bring the family back later in the year to catch up on further information. It’s really interesting, often when I do that by the time I bring the family back they’ve been on Facebook and found 12 families with the same condition and they know more than me about it! But, yes, so providing an opportunity, if you can, for them to come back. (UK clinical geneticist)

Health professionals identified a number of barriers to providing follow-up care, including limited resources (i.e. workforce shortages, commissioning), practical and/or legal restrictions on the use of telehealth (e.g. email and on-line consultations), need for innovative service models to deliver post-test care (e.g. collaboration with non-genetic health professionals), the shorter times of result appointments compared to consent appointments, and the lack of flexibility in booking appointments.

### Family interviews and feedback events

A total of nineteen families took part in the filmed interviews, including twenty-three participants (*n*=13CZ; *n*=10UK). Of these, eleven participated in the feedback events together with three patients and six members of the family advisory groups for a total of twenty participants (*n*=12CZ; *n*=8UK). Two families, one at each site, withdrew after the interview phase for changes in their personal circumstances. Their data was not used in the following stages of the study and is not reported here.

The key touchpoints emerging from families’ interviews were broadly similar across the two sites, with all participants reporting benefits from taking part in genomic testing (see Table 3 for a summary of the comparative analysis). A total of ten priorities were identified during deliberation at the feedback events. While some of these reflected issues specific to each site, both cohorts identified follow-up care as a key priority for improvement. Families at both sites highlighted how the emotional impact of receiving the results, and the amount and complexity of the information that was shared during the appointment, could leave them feeling ‘overloaded’ and generally overwhelmed. Families focused on taking in as much as possible during the consultation, but struggled to ask questions, as corroborated by our observations. This meant that some of the questions that were more pressing for families (e.g. What does it mean for us? What can we expect?) could be difficult to address during the appointment, as illustrated by the following excerpt (UK3):

Father: But you know, there might not be any reference to [older patients] at the moment but it might come that there might be a reference to them at that age, so then we can say ‘well yeah, in ten years’ time our daughter might be at this stage or this might be happening, which you don’t know, do you?

Interviewer: Is it something that you discussed with the geneticist at the appointment, and do you remember what she explained to you about what you can expect?

Mother: No, we didn’t.

Father: We never really went into what to expect because the day we went and got the diagnosis, which was the last time we saw the geneticist, I think we got overloaded with what we got told that day.

Mother: In the nicest possible way.

Time was needed for families to process the new information and to reflect on the implications of the results. However, families often felt that following the appointment, they had nowhere to go to discuss possible questions and concerns:

[I feel] lost. Because there’s information I need and there is no...It’s almost like somebody giving you the diagnosis but then going away and then having, say, school asking questions and I’m like ‘I’ve no idea.’ (UK2)

The lack of disease-specific information on new diagnoses was highlighted as an issue. As a result, participants struggled to use the results to improve care for their child and to access the support they needed:

Right now, I’m looking for a GP for adult patients. I was told that it would be very hard to find a doctor for a child with multiple physical and psychological problems...It is difficult to find any doctor or specialist, let alone finding a doctor who knows something about it and who cares, someone who is not resigned or won’t say ‘I don’t know what to do with this child.’ Recently, at endocrinology I was told they were surprised we were even referred to them because they don’t know what to do with these patients. (CZ3)

From this perspective, the experience of newly diagnosed families was not too dissimilar to that of families who received inconclusive results. This latter group often struggled to find information about VUSes and possible next steps available to them (e.g. further research and/or re-analysis once new information would become available in international databases). At the same time, families who received null results were often appreciative of the care received despite the lack of a diagnosis.

### Joint events and co-design process

Twenty-six members of staff (*n* = 17 CZ; *n* = 9 UK) and nineteen family participants (*n* = 11 CZ; *n* = 8 UK) took part in the next stage of the EBCD process. Reflecting the outcomes of the previous events, post-test care was voted as a shared priority for improvement at both sites. The focus was particularly on supporting newly diagnosed families to process and make use of the new information. At the UK site, participants’ ideas converged on co-designing a dedicated role (i.e. Liaison Officer), which was modelled on an existing role within the service. The Liaison Officer would serve as a first point of contact for families, facilitating follow-up engagement with the service and signposting them with external support as necessary. It would be accessible through self-referral to families at all stages of their journey, irrespective of whether they received a diagnosis. In the case of a newly confirmed diagnosed families, it would provide short- and medium-term emotional and practical support to facilitate families using the new information. At the Czech site, participants’ ideas focused on the provision of follow-up appointments with the clinical geneticist who had initially returned the results. This was offered to all newly diagnosed families; families who received inconclusive or negative results continued to remain in the care of the genetic department and were judged on a case-by-case basis.

Both interventions were underpinned by broadly similar design principles, including the following:

Family-centred approach: the interventions aimed at creating opportunities for families to explore questions such as ‘what does this mean for us?’ and ‘how can we use this information to improve our situation?’. As discussed, due to the specific nature of genomic testing, the implications of results for the individual family cannot be fully anticipated and discussed pre-test. Further compounding the uncertainty of genomic results is the lack of disease-specific information and established care pathways related to the new diagnoses. These factors meant that, even when a new diagnosis was confirmed, families required on-going support to their unique needs.

Active family participation: as noted, consultations when results are shared tended to be clinician-led and families often struggled to actively take part in the discussion due to the emotional impact and complexity of the new information. Allowing time for families to reflect on the results and carry out their own research was therefore considered essential to enable them to formulate and raise their own questions. At the UK site, this leads to the establishment of a dedicated role to facilitate family-led engagement with the service following the communication of the results. At the Czech site, participants highlighted the importance of trust and the existing relationship with the consulting clinical geneticist, converging towards a model of communication in two stages: one focused on clinician-led explanation of the results, and a follow-up discussion centred on family’s questions.

Focus on psychosocial and community support: at both sites, family participants felt that the clinical implications of results for the patients and other family members were satisfactorily addressed during the consultation. However, rare diseases are chronic and require continued support across social and community services (Castro et al. 2017). Positive results confirming a new diagnosis can impact the family’s position with regards to applying for benefits, accessing services and researching new information about the condition. Even when results are inconclusive, services with specialist expertise on rare diseases can play an important role in sign-posting families to available sources of support, such as generic support groups for rare (including undiagnosed) diseases. Interventions at both sites therefore aimed to improve the link between diagnostics (whether a diagnosis was confirmed or not) and referral to social and community services. At the UK site, this was considered the core component of the intervention, leading to the design of a non-clinical role of link worker. At the Czech site, participants decided that relevant information, including on patients’ organisations for rare diseases, should be included in the letter provided to families following the appointment.

### EBCD process evaluation

In this final section, we provide an evaluation of the EBCD process based on participants’ survey responses and our experience of delivering the EBCD process in this setting. We conclude with a review of practical challenges and recommendations for future projects using similar methodologies.

#### Participants’ experience

The evaluation surveys were complemented by 18 participants at the health professional events (8 UK and 10 CZ); 14 participants at the family events (7 UK and 7 CZ) and 46 participants at the joint events (including 10 UK and 12 CZ HPs; and 6 UK and 8 CZ family participants). At both sites, events were rated positively by both groups of participants, with feedback ranging from good to excellent. Overall, participants expressed positive feedback about the dynamics between participants and facilitators, and felt that they had the opportunity to actively participate in the discussion. The need to allow more time for deliberations was the most often cited suggestion to improve the format of the events.

In the open-ended questions, participants highlighted the value of co-production and the focus on lived experience. Health professionals appreciated the ‘rare opportunity’ for discussing such matters with colleagues. One health professional from the UK site described the events as ‘an opportunity for innovation’, while another judged them as ‘relevant to everyday practice’. Families expressed similar views, as noted by one of the participants: ‘each family has its own experience and sharing them helps to mutual discussion’ (CZ family participant). Participants from both groups noted that the joint event helped them to get a fuller picture of their own experience. Health professionals showed strong interest in the film (see the ‘Trigger film’ section’). Family participants also valued the opportunity to find out more about the work that goes on behind the scenes at the services and ‘how professionals tried to negotiate the complexities involved in implementing change’ related to the rollout of new genomic testing (UK family participant).

#### Trigger film

The film was effective in representing the broad range of family voices, triggering discussion and addressing possible power imbalances at the events. At both sites, participants found that overall the touchpoints included in the final edit provided a good representation of the main issues which families face. Health professionals found it impactful to hear directly from families, with participants describing it as ‘thought provoking and moving’ (UK participant) and ‘interesting for every clinical geneticist’ (CZ participant). Interestingly, families at the both sites also made similar comments, noting that the film helped them to appreciate the wide range of diverse experiences that other families can face.

In line with findings from previous EBCD studies (née Blackwell et al. 2017), we found that the film was a valuable resource for dissemination and engagement activities beyond the scope of the study, including as part of accelerated EBCD (Locock et al. 2014). Following the events, members of staff at the both sites have expressed interest in showing the film during departmental meetings, using it for training sessions or even during clinical genetics courses for medical students. The patient organisations which supported the study at the UK and Czech sites also expressed their interest in using the film as part of their engagement and educational activities with health professionals from other services. The film has also been shared during presentations with a variety of audiences, including rare disease families and advocates, and was found to be a powerful trigger for discussion.

#### Local quality improvement interventions

The large majority of participants reported that the priorities selected at each event reflected their experience of the respective service and their views on how these could be improved. There were, however, some exceptions, as it is to be expected given the heterogeneity of participating families. The implementation of the quality improvement interventions that were co-designed as a result of the study was not uniform, as the timeline of the design process and resources needed varied for each intervention (see Table 5 for details on each prototype).

For instance, at the Czech site, the main quality improvement intervention (i.e. follow-up consultation) started to be implemented as part of the study. Adjustments were made to the schedule of clinical appointments to provide follow-up appointments to families who recently received a new diagnosis. To address the second priority (i.e. managing feelings of guilt), a workshop on the psychological aspects of the communication process was designed in collaboration with two clinical psychologists collaborating with the organisation Rare Diseases Czech Republic. The workshop took place in March 2020, just before the Covid-19 pandemic restrictions were implemented. It was attended by clinical geneticists from the participating service, as well as other genetic services, and was positively evaluated by participants. To address the third priority (i.e. improvement of the physical environment of the service), members of staff designed a survey to gather the views of all patients and families visiting the service and identify key areas of improvement (e.g. improvements to the waiting room). Unfortunately, due to subsequent reprioritisation of university hospital services due to Covid-19, these infrastructural plans could not be implemented.

At the UK site, it was not possible to secure funding to pilot the new role of Liaison Officer within the timescale of the project. To address the other priority identified by participants, an educational intervention was prototyped, which comprised key principles and practical recommendations to improve communication at the point of testing. Plans were discussed to pilot the intervention as part of existing engagement and educational activities delivered by the service, including with trainees and non-genetic health professionals. The delivery would include using the short film from families interviews as an aid to convey the impact of expectation management on family experience. As with the Czech site, plans had to be put on hold due to Covid-19 and the disruptions to the work of the service.

#### Reflective assessment of challenges and recommendations for future projects

We found that there were a number of considerations to keep in mind in order to effectively deliver the EBCD process in this type of setting. First, limited time and resources represented a major issue at both sites. EBCD projects are typically completed within 12–18 months; however, delays in obtaining initial ethical approvals and completing the recruitment of family participants affected the project timeline. While this is not unusual, it affected the time available for piloting and evaluating the co-designed interventions. Resource issues also meant that some of the priorities that participants considered most urgent could not be addressed, and not all quality improvement interventions could be fully implemented (e.g. as noted, at the UK site, it was not possible to create a new position of Liaison Officer within the time and resources of the project). This is not uncommon in the case of EBCD, which is more likely to initially lead to incremental quality improvements rather than radical organisational change (Donetto et al. 2014). Future projects should consider these issues at the design stage (i.e. allowing adequate time and resources for implementation and evaluation of EBCD-based improvements), while also managing participants’ expectations as to what can and cannot be achieved through the process.

Second, facilitating effective participation from families can present a series of challenges (Oliver et al. 2019). In our case, we found that these practical challenges were in part exacerbated by the very heterogeneous presentation of rare diseases, and the different implications that each type of results (i.e. new diagnosis, variants of unknown significance and null results) can have for individual families. While participants’ feedback converged around issues of wider relevance (e.g. post-test care), more specific issues could not be fully addressed. Participants’ specific needs and expectations need to be carefully considered and managed in the process of negotiating shared priorities so that the process is conducted in a way that does not feel exploitative or disappointing.

Third, it should be noted that, as a participatory methodology, EBCD can require significant time and investment from participants, for example, to travel to events and take part in meetings. This might be challenging to reconcile with professionals’ commitments and families’ caring responsibilities. Providing practical support for participants to attend is key to mitigate against possible bias related to self-selection of participants and ensure inclusiveness and representativeness. Specifically, it is important to ensure a balance between valuing the expertise of patients, as developed through previous engagement with research and advocacy work, and supporting the involvement of those who might not have had previous exposure to similar activities (Prainsack 2014; Zagarella and Mancini 2020).

Fourth, despite everyone’s best effort, we encountered some practical challenges in developing a sense of ownership and investment in the project across all stakeholders. EBCD is often used for internally initiated processes of quality improvement. In our case, the project was initiated and delivered by external researchers (in collaboration with service staff). This meant that researchers’ time on site was, inevitably, sporadic. This could affect access for observations, meaning it could be challenging to develop a rounded view of routine work at the service beyond the delivery of individual clinical appointments. Ensuring active participation from members of staff at the local services could also be challenging, due to conflicting commitments. Strong leadership from senior staff was a key aspect in our experience. On-going communication about the project, for example, through internal emails and during team meetings, is also essential to reach out to members of staff who might not have the opportunity to come in direct contact with the researchers, and to articulate and embed the aims of the project within the routine work of the service.

Finally, the project was initiated and conducted as part of the larger Solve-RD research study with the aim of producing and disseminating findings that can be adopted in other settings. This distinctive research focus meant that, in the co-design process, there were at times tensions between the development of quality improvement interventions that could be implemented immediately at the level of the individual services, and the identification of co-design principles that could be generalised and scaled up to different settings. While this is not an issue per se, future researchers using similar methods should be mindful of such distinctions and their implications.

## Discussion

The communication of genomic results raises distinctive challenges for health professionals and rare disease families. Evidence is gradually emerging on patients’ and families’ understanding of and attitudes towards genomic testing (Lewis et al. 2020; Genetic Alliance UK 2015; Halverson et al. 2016; Wynn et al. 2018b; Mollison et al. 2020; Watnick et al. 2021) as well as on health professionals’ experiences of returning results (Ormondroyd et al. 2017; Wynn et al. 2018a). This growing body of research has provided important insights into communication strategies to facilitate families’ comprehension of and engagement with genomic information in clinic (Williams et al. 2018; Biesecker and Lewis 2021). Expanding on this evidence, in this study, we used EBCD to translate participants’ experience into quality improvement interventions, engaging participants in the process of co-designing models for the communication of results that best meet their needs. Through the process, families and health professionals identified a total of four priorities for improvement and co-designed six quality improvement interventions. The key finding was the identification of post-test care as a shared priority for improvement.

At both sites, participants highlighted how the emotional impact of the results, and the complexity of the information shared, meant that during the consultation families focused on taking as much as they could and had little space to formulate their own questions. Once they had the chance to reflect on the implications of the results, however, they lacked opportunities to follow up and raise questions. Families also reported facing enduring issues in using the new information to identify, apply for and access the right kind of support, even when a new diagnosis had been confirmed. The disjointed nature of care pathways for rare diseases (Tumiene and Graessner 2021) and the lack of disease-specific information and treatment, especially for the new and ultra rare diagnoses that genomic testing is gradually making possible to confirm (Rosell et al. 2016), emerged as key issues, pointing to the need of continued support across health, as well as social and community services (Castro et al. 2017). Improving post-test care was therefore considered essential to realise the benefits of genomic results in practice. This, however, remained challenging under the current genetic service models.

These findings present two important considerations in relation to current and future models of genomic health services. The first relates to the need to rethink current genetic service models and the configuration of professional roles and responsibilities (Battista et al. 2012). Post-test care has been identified as a pressing issue in relation to future models of genomic services, calling for a shift in focus from just improving access to testing towards providing support for families in dealing with the consequences of testing (Patch and Middleton 2018). Addressing these issues, the findings start to fill the gap in empirical research on key components of post-test care in need of improvement, as identified by families and health professionals. Specifically, commissioning was highlighted as a problem at both sites, pointing to the need to direct more resources towards post-test appointments. Another issue identified was the need to bridge the gap between diagnostics (whether or not a diagnosis is established) and post-test care through the provision of medium-term support centred around the family’s unique needs. Genetic counselling can provide effective psychosocial support and empower families to use genetic information; however, genetic services remain grossly understaffed (Jenkins et al. 2021), meaning that engaging the wider workforce will be key to deliver the benefits of genomic medicine. In particular, the findings point to the need to strengthen the links with social care and community services (Castro et al. 2017; Simpson et al. 2021; EURORDIS 2021), through non-clinical roles (e.g. Liaison Officer) and innovative models for integrated care.

The second implication of the findings relates to the question of family and health professional involvement in research. As mentioned, the literature on families’ and health professionals’ experiences of sharing and receiving genomic information is growing, but studies are scarce that draw on this evidence to involve participants in service design and quality improvement. Researchers and patient advocates have long highlighted the importance of promoting active engagement of rare disease patients and their families with research. However, to date, efforts have mainly focused on involvement in selected aspects of medical research. Patients and families tend to be consulted unilaterally in relation to priorities pre-defined by researchers most commonly to improve recruitment into trials and identify patient-centred outcomes (Forsythe et al. 2014). Their involvement is rarely sought at the agenda-setting and translational stages, if not to support the dissemination of findings (ibid.). Our research shows that, while essential, improving diagnostics and treatment is only the start of the process of improving patients’ and families’ quality of life. Engaging families and health professionals in translational research, and in particular in health care research, will be key to ensure that new genomic knowledge can be used effectively to meet their needs. To this end, the findings presented in this article can be drawn upon and adapted to deliver the EBCD process in other settings to involve patients and families in the design and improvement of genetic/genomic services.

## Limitations and implications for future research

Not all the proposed quality improvement interventions could be implemented and comprehensively evaluated as part of this study. While the findings provide a strong rationale for the interventions, further evidence is needed to refine and adapt the prototypes in different settings. This might involve exploring health professionals’ experiences and needs more broadly, including in non-genetic settings; defining the needs of different groups of service users (e.g. families who might benefit from additional support following the return of results) and/or evaluating the implementation process in practice to identify possible challenges to integrate the interventions in routine clinical work.

## Conclusion

To the best of our knowledge, this was the first study to use the EBCD methodology with families of rare disease patients in the context of genomic health services (Donetto et al. 2014). Our findings highlighted the need of improving post-test care as an integral part of future genomic health service, and the importance of involving rare disease families and health professionals as active participants in the process of service design and quality improvement. The immediate contributions of genomics to health care are likely to be the greatest in the field of rare diseases. Improving diagnostics remains essential, but will not, in and of itself, suffice to maximise the benefits of genomic testing in medical care. Importantly, involving affected families together with health professionals will be crucial to the design of future genomic services that best meet their evolving needs.

## Footnotes

1 The name of this ERN represents a metaphor for the diagnostic odyssey that many families with rare diseases undergo (https://ern-ithaca.eu/).
