# Correction to: Co‑designing models for the communication of genomic results for rare diseases: a comparative study in the Czech Republic and the United Kingdom

**DOI:** 10.1007/s12687-022-00592-1

**Published:** 2022-07-01

**Authors:** Alessia Costa, Věra Franková, Glenn Robert, Milan Macek, Christine Patch, Elizabeth Alexander, Anna Arellanesova, Jill Clayton‑Smith, Amy Hunter, Markéta Havlovicová, Radka Pourová, Marie Pritchard, Lauren Roberts, Veronika Zoubková, Alison Metcalfe

**Affiliations:** 1 Engagement and Society, Wellcome Connecting Science, Hinxton, Cambridgeshire, CB10 1SA UK; 2 Faculty of Nursing, Midwifery and Palliative Care, King’s College London, London, SE1 8WA UK; 3 Department of Paediatrics and Inherited Metabolic Disorders, Charles University, First Faculty of Medicine and General University Hospital, Prague, Czech Republic; 4 Institute for Medical Humanities, Charles University, First Faculty of Medicine, Prague, Czech Republic; 5 Department of Biology and Medical Genetics, Charles University, Second Faculty of Medicine, and University Hospital Motol, Prague, Czech Republic; 6 Genomics England, London, EC1M 6BQ UK; 7 Manchester Centre For Genomic Medicine, University of Manchester, St Mary’s Hospital, Manchester, M13 9WL UK; 8 Česká asociace pro vzácná onemocnění (ČAVO), Rare Diseases Czech Republic, Bělohorská 19, Prague 6, 169 00 Czech Republic; 9 Division of Evolution and Genomic Sciences School of Biological Sciences, University of Manchester, Manchester, M13 9PL UK; 10 Genetic Alliance UK, London, EC2A 4NE UK; 11 Syndromes Without A Name (SWAN UK), London, EC2A 4NE UK; 12 Zinc Ventures, London, UK

**Correction to: Journal of Community Genetics**

**https://doi.org/10.1007/s12687-022-00589-w**

The original version of this article unfortunately contains mistakes introduced during the publishing process.

Tables 2, 3, 4 and 5 should be presented as below.

**Table 2:** Themes from interviews with health professionals and discussion at the events

Czech site	UK site
**Post-test care:** need to follow up with families after results have been shared, and recommendations for improvements in this area	
**Telehealth**: improve accessibility to facilitate communication with families, including via email and/or digital consultation	
**Multidisciplinary approach**: improve collaboration with non-genetic specialties as well as with allied health care professions (e.g. integrated service models).	
**Education:** about genomics in general and rare disease in particular, particularly among non-genetic specialties and in collaboration with patient organisations.	
**Counselling skills**: psychosocial support on challenging aspects of the family journey (e.g. expectation management, valuing negative results, managing feelings of guilt)	
**Lab reports**: accessibility of language and content of the reports for families and non-genetic professionals.	
**Family-facing educational and information materials** (e.g. improvements to service website)	**Resources** (e.g. workforce shortages, commissioning)
**Service environment** (e.g. wheelchair access, a suitable waiting room, a feeding and changing room for babies and toddlers)	**IT and datasharing** (e.g. patient database)

**Table 3:** Touch points from family interviews

Czech site	UK site
**Personal utility**: benefits families identified, including but not limited to the clinical utility of results (e.g. psychological benefits, benefits to other family members and future patients)	
**Making sense**: the emotional impact of results and information overload at the consultation meant that time was needed to process the implications of the results.	
**Unmet needs**: following the communication of results families often reported having unanswered questions and experiencing challenges in using the new information to improve their care, even when a diagnosis was confirmed.	
**Feelings of guilt and blame**: families’ sense of responsibility about causing the patient’s disability and/or passing on the conditions, which could be induced and/or exacerbated by the results	**Communication at the point of testing**: lack of openness and transparency about the reasons for testing, the different types of possible results and the impact on family’s expectations
**Service environment**: insufficiently spacious offices for large families, lack of barrier free-access and child-friendly spaces	**Communication about availability of results**: issues related the communication to inform families that the results are ready, including lack of notice, provision of impartial information and/or long waiting times for appointment, lack of consultation on family preferences

**Table 4:** Priorities for improvement

Czech site	UK site
Health professional priorities	
Post-test care: follow up with families after results have been shared Family-facing educational and information materials: provide resources and content of the service website Lab reports: improve accessibility by and utility for families	Post-test care: facilitate communication after results have been shared (e.g. telehealth) Multidisciplinary collaboration: information that can be used by non-genetic professionals (e.g. at the point of testing) Lab reports: clear and standardised reports to improve accessibility by non-genetic professionals
Family priorities	
Post-test care: follow-up consultation Psychosocial support: involvement of psychologist and/or social worker at results delivery Information provision Manage feelings of guilt and blame Improvement of service environment	Communication at the point of testing: transparency and expectation management Post-test care: support and advice after results are shared Post-test care: named point of contact Communication about results availability Multidisciplinary care: better coordination between genetic and non-genetic professionals
Shared priorities	
Follow-up consultation Managing feelings of guilt and blame Environmental improvements	Communication at the point of testing: transparency and expectation management Named point of contact for follow up

**Table 5:**
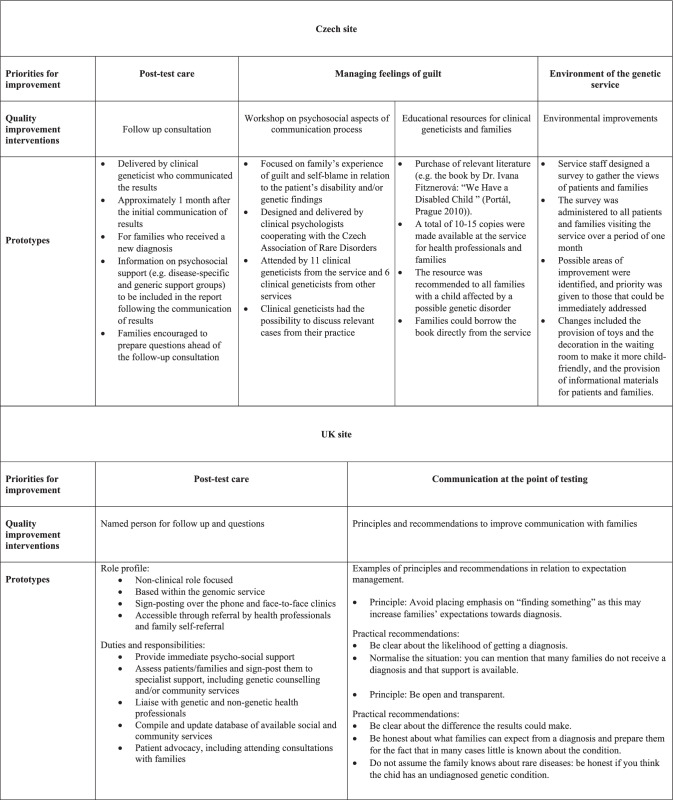
Quality improvement interventions at the two sites

This is being corrected in this publication.

